# The Formation of Antibiotic Resistance Genes in Bacterial Communities During Garlic Powder Processing

**DOI:** 10.3389/fnut.2021.800932

**Published:** 2021-12-16

**Authors:** Yanxia Liu, Peng Gao, Yuhao Wu, Xiaorui Wang, Xiaoming Lu, Chao Liu, Ningyang Li, Jinyue Sun, Jianbo Xiao, Simal-Gandara Jesus

**Affiliations:** ^1^Key Laboratory of Food Processing Technology and Quality Control in Shandong Province, College of Food Science and Engineering, Shandong Agricultural University, Taian, China; ^2^Key Laboratory of Novel Food Resources Processing, Ministry of Agriculture and Rural Affairs/Key Laboratory of Agro-Products Processing Technology of Shandong Province/Institute of Agro-Food Science and Technology, Shandong Academy of Agricultural Sciences, Jinan, China; ^3^Nutrition and Bromatology Group, Department of Analytical Chemistry and Food Science, Faculty of Science, Universidade de Vigo, Ourense, Spain

**Keywords:** garlic powder, microbial community, antibiotic resistance genes, metagenome sequencing, co-occurrence patterns

## Abstract

Chinese garlic powder (GP) is exported to all countries in the world, but the excess of microorganisms is a serious problem that affects export. The number of microorganisms has a serious impact on the pricing of GP. It is very important to detect and control the microorganism in GP. The purpose of this study was to investigate the contamination and drug resistance of microorganisms during the processing of GP. We used metagenomics and Illumina sequencing to study the composition and dynamic distribution of antibiotic resistance genes (ARGs), but also the microbial community in three kinds of garlic products from factory processing. The results showed that a total of 126 ARG genes were detected in all the samples, which belonged to 11 ARG species. With the processing of GP, the expression of ARGs showed a trend to increase at first and then to decrease. Network analysis was used to study the co-occurrence patterns among ARG subtypes and bacterial communities and ARGs.

## Introduction

Antibiotic resistance genes were first proposed by American scholar Pruden (1) in 2006. Antibiotics are persistent in environmental media and spread between different hosts. They pose a threat to public health and food safety and have become of keen interest in various fields such as botany, soil science, and food science (2). The increase in the antibiotic resistance of bacteria (ARB) infection and ARGs from antibiotics (3, 4), or other substances [including heavy metals (5, 6), light irradiations (7), or mineral nanoparticles such as zinc (8)], of selective pressure by horizontal gene transfer (HGT), appeared in the emerging transmission, also has caused great attention all over the world. Since their discovery in the 1930s, antibiotics have been widely used and abused as their availability has increased. ARGs can be transferred horizontally or present in microorganisms *via* mobile genetic elements (MGEs) including transposons, plasmids, integrons, and common regions of insertion sequences (9). Antibiotics not only pollute the environment and threaten people's health, but also threaten the achievements of modern medicine. HGT promotes the spread of ARGs among different types of microorganisms. This enables more microorganisms, especially pathogenic bacteria, to acquire resistance, and leads to the emergence of multiresistant bacteria (10–12). Studies have shown that antibiotic resistance causes more than two million diseases and hundreds of thousands of deaths each year. The situation will get worse if action is not taken; the economic impact of uncontrolled antibiotic resistance will be catastrophic (13, 14). An assessment of the repository of ARGs and environment persistence is essential to implement a strategy to reduce the propagation of ARGs.

To date, numerous experiments and studies have been conducted to uncover the presence and abundance of ARGs in media as landfills, industrial and medical wastewater, and livestock farms and soils (15). Soil is one of the habitats with the highest microbial diversity on earth, a source of natural antibiotics, and a repository of ARGs (12). Long-term abuse of antibiotics is likely to lead to the generation of ARGs in animals, which can pollute the environment and soil through excretion. They can also be spread widely through viruses and bacteria, which results in a series of genetic contamination and food safety problems. Some reports showed that a high abundance of beta-lactam resistance genes was detected in food waste-recycling wastewater, and the ARG pollution status in food wastes was studied (16). BlaOXA-20 was detected on vegetables such as radish and lettuce grown in sludge modified soil, and the relationship between resistance genes and pathogenic bacteria in vegetables was studied (17). There have been reports of risks of resistant microbes in vegetables (such as tomatoes, celery, and radishes), fruits (such as pears and strawberries), meat, and seafood (18, 19), which suggests that food may be a carrier of ARB or ARG dissemination to people through the food chain, which also poses a new challenge to food safety (20, 21).

Garlic has attracted more and more attention for its nutritional value and functions. China is the largest grower and exporter of garlic in the world. Garlic is also one of the important and economical crops in China. Garlic is highly seasonal, so it is usually processed into various garlic products, such as dehydrated garlic slices (GS), frozen garlic, garlic powder (GP), garlic oil, and so on, to enhance its availability. As a flavored food, GP retains all the original ingredients of garlic except water, including alliin and the active alliinase. It not only can improve food flavor and appetite but also can be processed into GP and used as a garlic supplement to play a certain effect, playing a key role in enterprises' research and development of new products (22). In recent years, China's condiment industry is growing at an average annual rate of about 20% with obvious social and economic benefits (23). The problem of microbial contamination in GP has not been well solved, which seriously affects the export of GP and other garlic products; the number of microorganisms will also seriously reduce the price of products. It is very necessary to study the microorganism of GP.

As the host of ARGs, microorganisms are closely related to the existence of ARGs. To determine the existence and abundance of microbial communities and ARGs in garlic products, increase public awareness of the potential risks of ARG contamination and dissemination, and provide potential indicators and host information for the ARGs, this article uses metagenomics and Illumina sequencing methods to study the distributions and similarity/difference in ARG compositions and dynamics of microbial communities in three kinds of garlic products. The relationship between ARGs and microbial communities was discussed and also investigated the co-occurrence patterns among ARG subtypes and microbial taxa in garlic cloves (GC), garlic flakes, and GP using network analysis. This article reveals the special relationship between microorganisms and ARGs in the processing of GP, fills the knowledge gap of microbial and antibiotic resistance in garlic products, and provides valuable clues for the removal of ARG in garlic products.

## Materials and Methods

### Sample Collection

The garlic products were randomly selected from a local garlic processing plant in Pizhou, Jiangsu Province of China. The rough processing process of GP in enterprises was as follows: garlic was peeled and split after passing the acceptance. After cleaning and removing the surface dirt and impurities, it was processed into GS for further color protection and drying. The thickness of GS was generally 3 mm, milky white or milky yellow, and the water content does not exceed 5%. GS was crushed by a grinder and processed into GP through an 80-mesh sieve. The sample for this experiment was taken from a local garlic product manufacturer, and three samples of GC without mechanical damage and physiological diseases were collected. GS and GP were processed products, both of which were sealed and packaged in sterile bags, and three samples of each were also collected. The samples were inspected and analyzed immediately after being delivered to the laboratory.

### DNA Extraction, Library Construction and Sequencing

The sample processing refers to the method of Sessitsch (24) and Huang (25) with slight changes. The collected GC samples were first rinsed with sterile water, then soaked in 70% ethyl alcohol for 2 min, immersed in 2.5% sodium hypochlorite for 5 min, and finally rinsed with sterile water three times. Then, 100 μl of sterile water from the final washing was taken and spread on PDA and NA medium for the epiphytic check. Samples without colony growth after culturing at 28 ± 2°C for three days were used for subsequent metagenomic sequencing.

The sample after the surface sterilization treatment was crushed in a mortar under a sterile environment placed in a 0.9% NaCl solution containing sterile glass beads, and shaken at 30°C for 4 h. Then, it was centrifuged at 6,000 g for 5 min to remove debris, and the supernatant was filtered through a 0.22-μm filter membrane (EMD Millipore, Temecula, CA). The filter membrane was cut into pieces and the DNA was extracted by the CTAB method and stored at −20°C until analysis.

The concentration and purity of purified DNA were detected by NanoDrop One instrument. For metagenomic sequencing, 1μg of DNA was taken to construct a sequencing library of 300-bp inserts, and then, the Illumina HiSeq 4,000 sequencing platform was used with the sequencing strategy of PE150 (paired-end sequencing 150 x 20).

### DNA Sequence Assembly and Annotation

First of all, the original off-machine data used Trimmomatic v0.39 (26) for quality control and the reads with an average quality value of <30 (*Q* ≤ 30) were removed to obtain a high-quality sequence. BWA v0.7.17-r1188 (27) was used to align the high-quality reads to the custom database of garlic genome (https://www.ncbi.nlm.nih.gov/assembly/ GCA_014155895.1) and to remove the host genome from the alignment sequence. The unaligned high-quality sequence was assembled using SPAdes v3.15.2 (28) to obtain scaffolds (length ≥ 500 bp). The reads were re-aligned back to the assembled sequence, and the indel and base-pair errors were corrected according to the depth of the alignment (29, 30). MetaGeneMark (30) was used to predict genes in assembled scaffolds, and CD-HIT (31) was used to cluster genes with 95% consistency and 90% repetition rate, and to remove redundant genes. In addition, the bedtools v2.29.2 multicov (32) program was used to calculate the relative expression abundance of each gene.

The antibiotic resistance database (Comprehensive Antibiotic Resistance Database, CARD) (33) contains a large number of known ARGs and their related resistant antibiotics. Subsequently, we aligned the metagenomic gene sequence to the CARD database to predict drug resistance. To ensure the accuracy of ARGs, an 80% consistency threshold was selected as the standard (34, 35).

Bacterial, archaeal, viral, and eukaryotic are distinct from each other in genomes. To determine the species classification information and the abundance of each species in the metagenomics, Kraken2 v2.0.9-beta (36) and Bracken (37) were used to analyze the nongarlic genome data after quality control. The reference database is the minikraken2-V2 database (released on April 25, 2019).

### Statistical Analysis

All statistical analyses considered that *p* < 0.05 was statistically significant. Principal component analysis (PCA) was used to assess the bacterial community and ARG profiles among different samples. Venn diagrams were drawn using Venn Diagram package v1.6.20 or UpSetR package v1.4.0, while heatmaps were generated with the heatmap package by v1.0.12. The relationship between bacterial communities and ARG subtypes and their respective internal relationships were evaluated by Spearman's correlation. Pearson's correlations were used to assess the relationships between gene functions and bacterial communities. Then, we removed the correlation in which the coefficient was below 0.90 and the *p*-value was above 0.01 and adjusted the *p*-value to avoid false positives using the false discovery rate (FDR) method (38). Network analysis and visualization were conducted with the Gephi v0.9.2.

## Results

### ARG Evolution Profiles During GP Processing

**Figure 1:**
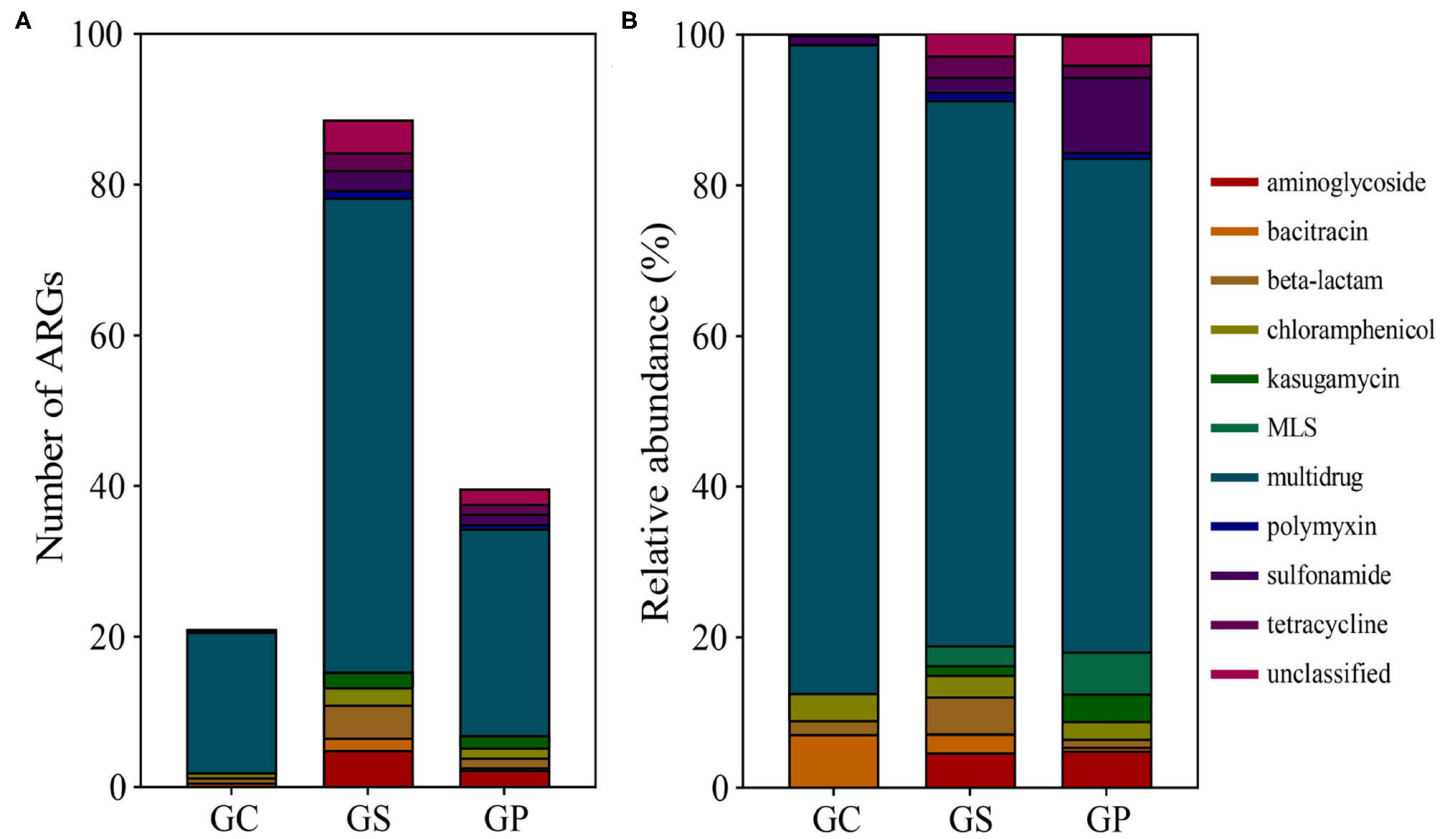
ARGs detected during GP processing. **(A)** The number of resistance genes detected during GP processing. **(B)** The relative abundance of ARGs in the three products.

A total of 126 ARGs were detected in all samples, which belong to 11 ARG types. Among them, 29 shared ARGs were common in the GP processing process, accounting for 23% of the total ARG. In addition, 53 (42%) ARGs coexisted in GS and GP (Supplementary Figure S1), indicating that there was a higher similarity in ARGs in the middle and late stages of GP processing. The average abundances of ARG in GC, GS, and GP were 21.0, 88.7, and 39.7, respectively (Figure 1A). The detection frequency of ARGs showed an upward trend in GS processing and a downward trend in GP processing, indicating that the GS processing would lead to an increase in ARGs, and further processing into GP would lead to a decrease in ARGs. Further analysis of the distribution of ARGs showed that multidrug was the ARG with the highest abundance in the three periods (Figure 1B), which were 86.14, 72.41, and 65.62%, respectively. Second, bacitracin (7.23%) and chloramphenicol (3.61%) were the main ARGs in GC, beta-lactam (4.96%) and aminoglycoside (4.75%) were the main ARGs in GS, and sulfonamide (9.97%), MLS (5.51%), and aminoglycoside (4.99%) were the main ARGs in GP.

**Figure 2:**
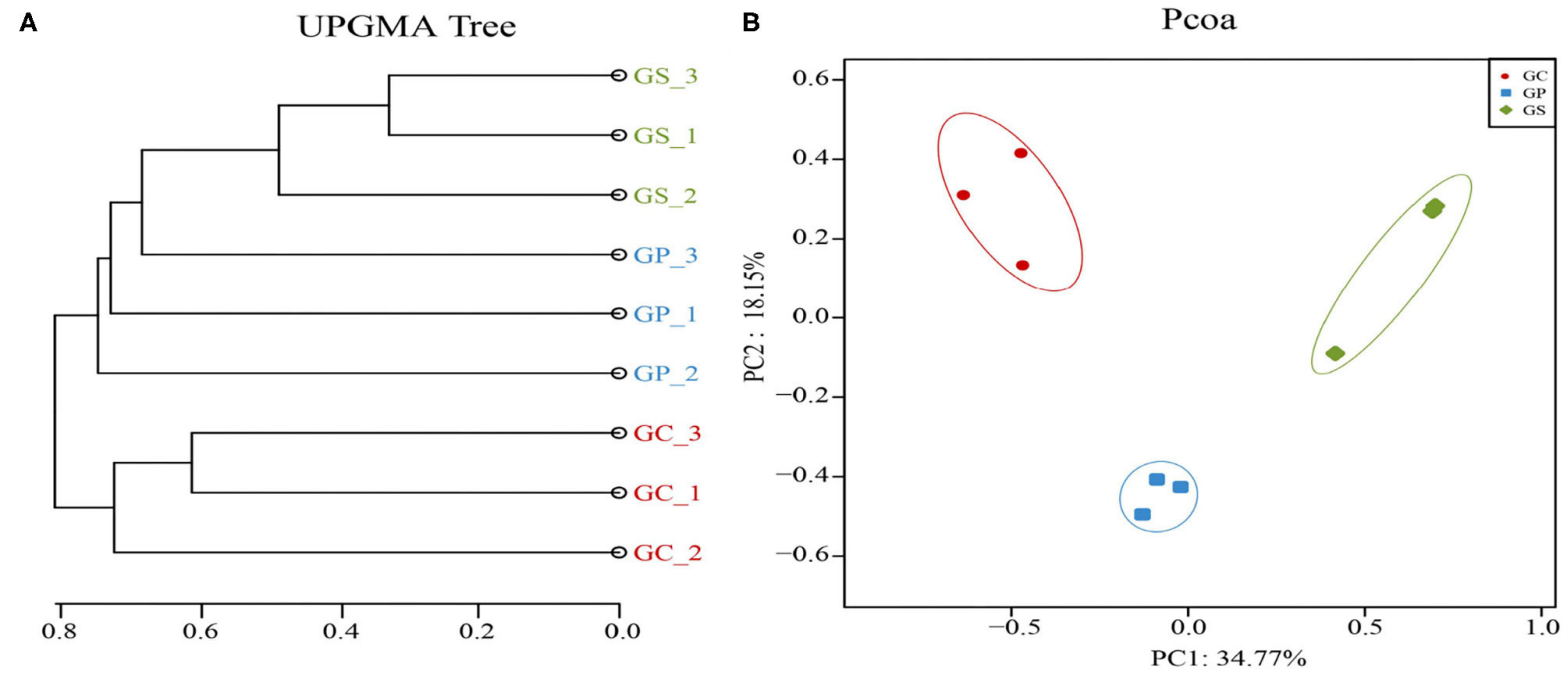
The differences in the composition of ARGs during the GP processing. **(A)** UPGMA cluster analysis of ARG abundance in three garlic products. **(B)** PCA based on Bray–Curtis distance showed the overall distribution pattern of ARGs during GP processing.

Unweighted pair group method with arithmetic mean (UPGMA) cluster analysis based on the abundance of ARGs and PCA analysis of Bray–Curtis distance further revealed the differences in the composition of ARGs during the GP processing (Figure 2). In the same process, ARGs clustered together and separated from other ARGs in the different processes. In addition, GP and GS were more similar in UPGMA clustering, and both were far away from GC. It showed that there were changes in the composition of ARGs in GP processing, and the changes in ARGs in the middle and late stages were more similar than those in the early GC.

**Figure 3:**
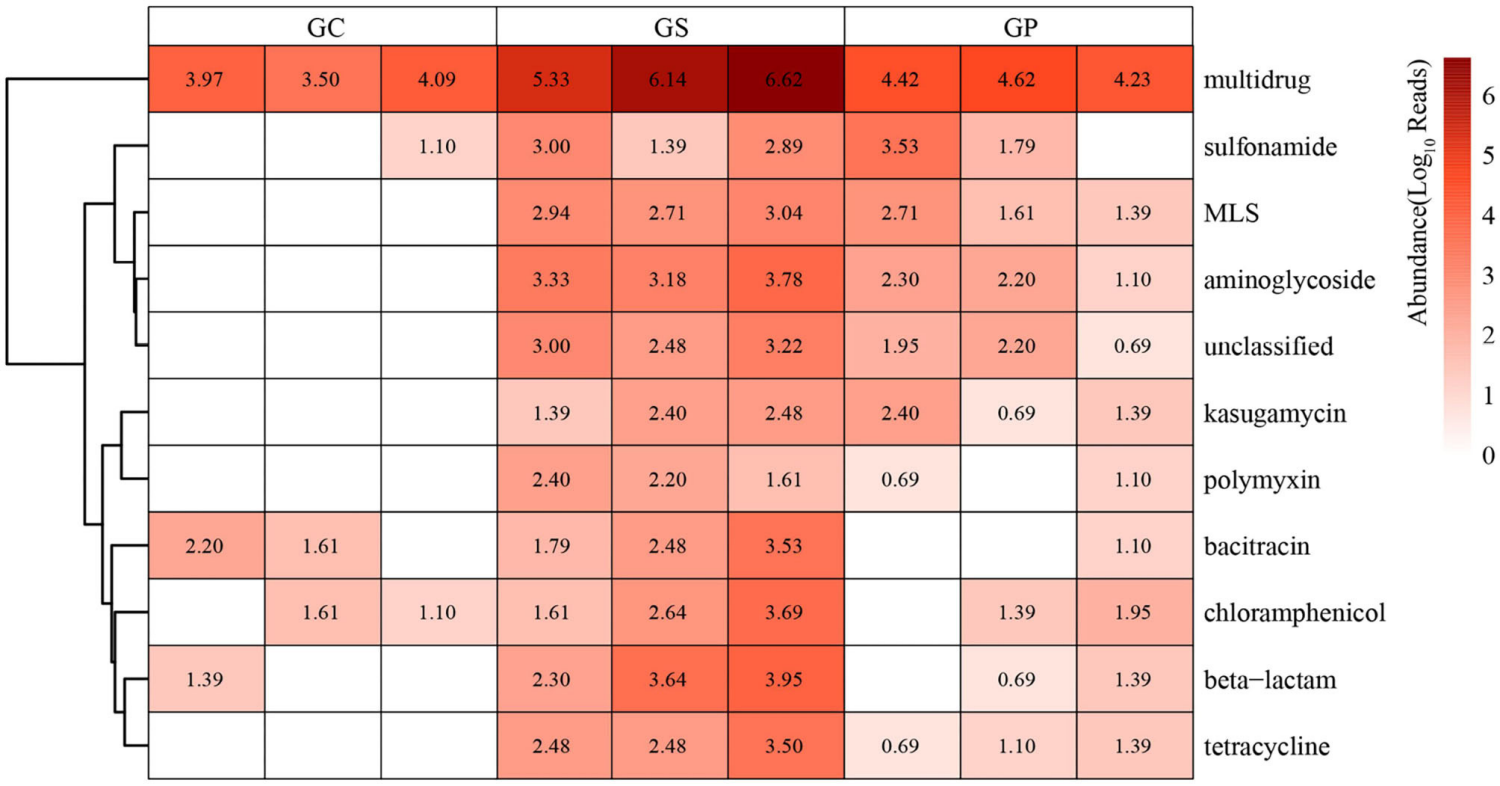
The distribution of ARGs. Heatmap showed the distribution of ARGs in different types of products during GP processing.

With the processing of GP, the performance of ARGs increased first and then decreased (Figure 3). Specifically for multidrug, mexB, mexD, mexW, smeE, etc. showed a trend of first increasing and then decreasing (Supplementary Figure S2). It showed that the processing from GC to GS would increase the abundance of ARGs such as multidrug, aminoglycoside, and MLS, and with the processing from GS to GP; the increased ARGs would decrease to varying degrees.

### Co-Occurrence Patterns Among ARG Subtypes

**Figure 4:**
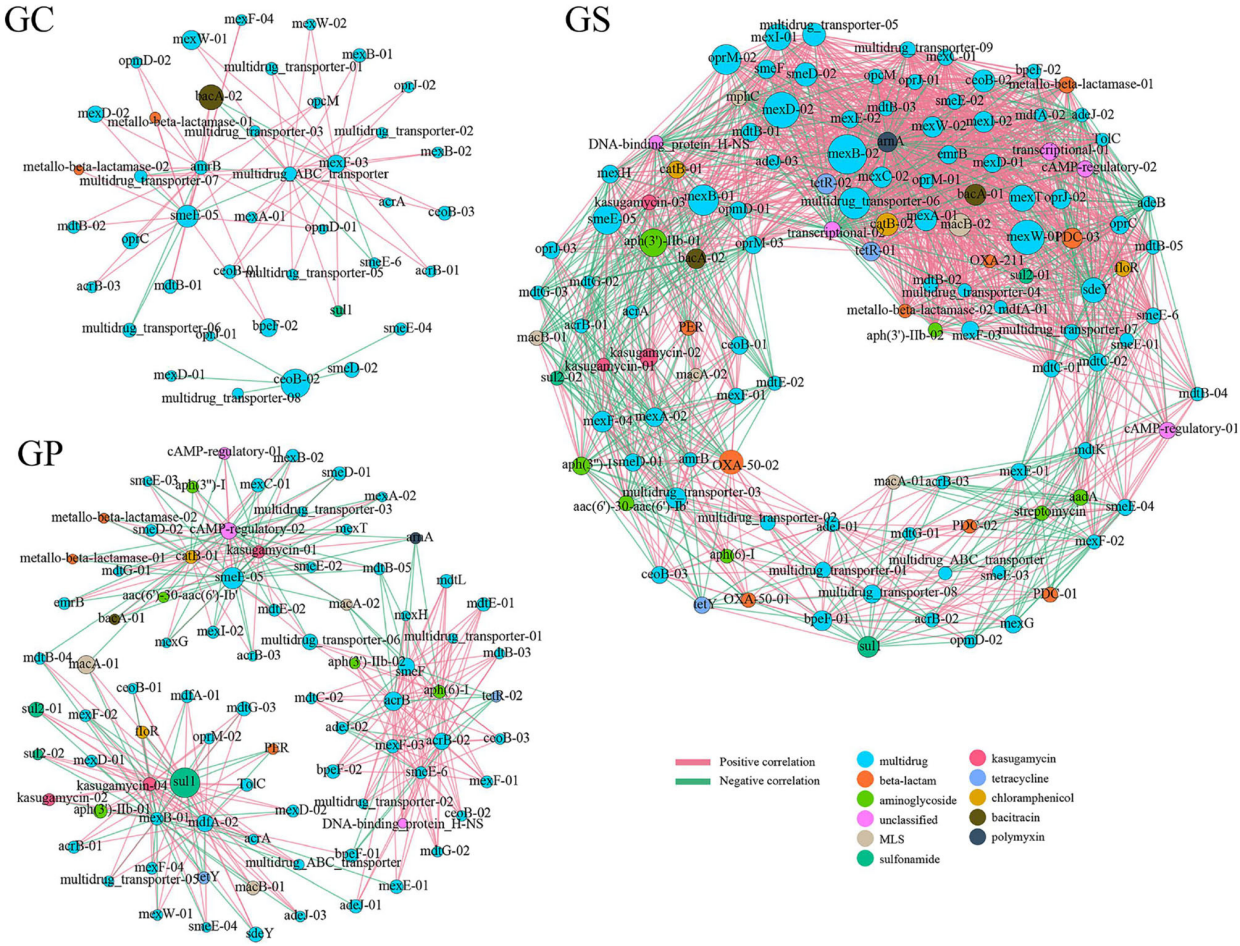
The network analysis revealing the co-occurrence patterns among ARG subtypes in GC, GS, and GP. The nodes were colored according to ARG types. A connection represents a strong (Spearman's correlation coefficient *r* > 0.90) and significant (*p* ≤ 0.001) correlation. Edges and node size weighted were based on the correlation coefficient and the relative abundance of ARGs, respectively.

Based on Spearman's correlation analysis (*r* > 0.9, *p* ≤ 0.001), the co-occurrence patterns among ARG subtypes were investigated using network analysis. As shown in Figure 4, the GC co-occurrence network contained 41 nodes and 75 edges. Among them, multidrug ABC transporter had an obvious correlation with other 21 ARGs (17 positive correlations and four negative correlations), and mexF-03 had obvious correlations with other 19 ARGs (17 positive correlations and two negative correlations). In addition, amrB and smeE-05 were also related to the other 17 ARGs, respectively. Compared with GC and GP, GP had a more complex co-occurrence network of ARGs, including 122 nodes and 2,100 edges. In particular, bacA-01, macB-02, and mexD-01 were correlated with 60 other ARGs, respectively, and the positively correlated ARGs contained 40 (66.7%), 40 (66.7%), and 39 (65%) multidrug, respectively. Genes belonging to multidrug in GS occupied 88 nodes (66.3%), of which mexD-01, mexW-02, mexB-02, multidrug-transporter-06, mexI-02, and mexE-02 were related to more than 57 ARGs, which were closely connected nodes, indicating that these genes could be used as indicator genes for ARGs. In addition, arnA (belonging to polymyxin) was negatively correlated with 52 other ARGs, including 38 multidrugs, four beta-lactams, two chloramphenicols, two tetracyclines, and so on. In GP, sul1 (belonging to sulfonamide) was located in the center of the co-occurrence network and had more complex correlations (30 edges) compared with other ARGs. Similarly, multidrug genes occupied 59 (69.4%) of the 85 nodes, of which mdfA-02, mexB-01, smeE-05, and acrB interacted with more than 25 ARGs, which were the main subtypes of ARGs in the network.

### Correlation of Bacterial Communities

**Figure 5:**
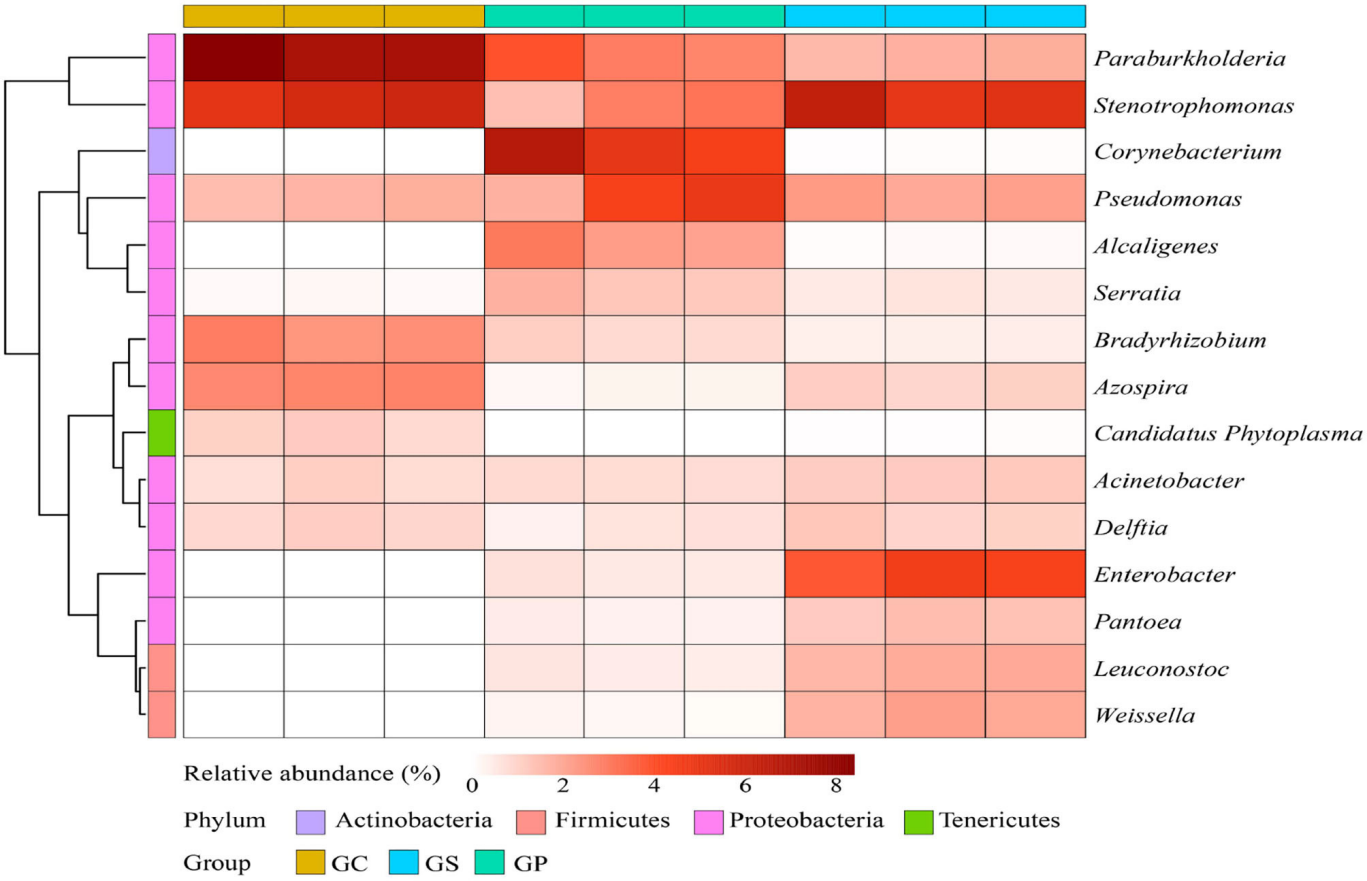
Heatmap of the relative abundance of bacterial communities at phylum and genus levels during the garlic processing. Clustered at the gate level according to abundance and similarity.

The transformation of microorganisms during GP processing was closely related to the types and abundance of resistance genes. It was necessary to explore changes in bacterial community composition during GP processing. Compared with GC, the GS and GP groups shared more genera (385), indicating that they might have a very similar community composition (Supplementary Figure S3). At the same time, the heatmap based on phylogenetic classification showed that the composition at the genus level exhibited different temporal changes during garlic processing (Figure 5). *Paraburkholderia, Stenotophomonas, Bradyrhizobium*, and *Azospira* were the four most dominant genera of GC in the early stage of GP processing. They all belonged to *Proteobacteria*, accounting for 18.67% of the total bacterial 16S rRNA gene sequence in GC. But in the middle and late stages of GP processing (GS and GP), the relative abundance of *Paraburkholderia, Bradyrhizobium*, and *Azospira* decreased rapidly (3.16%). After the relative abundance of *Stenotrophomonas* in GS decreased, it increased in GP again (the relative contents of GC, GS, and GP were 5.65, 2.50, and 5.59%, respectively). *Alcaligenes, Serratia*, and *Pseudomonas* were also three genera belonging to the phylum *Proteobacteria*. In the GP processing, it rapidly increased from 1.82% (GC) to 7.61% (GS), but it returned to a low content level (2.76%) in the later stage of the processing. *Corynebacterium* of *Actinobacteria* showed the same trend. *Leuconostoc* and *Weissella* belonged to the *Firmicutes* phylum and *Enterobacter* and *Pantoea* belonged to the *Proteobacteria* phylum increased in GP content (9.43%) in the later processing stage and were at low levels (0.01 and 1.58%) in the early and middle stages.

Strong competition and interactions existed in bacterial communities for limited resources during food processing. Network analysis was employed to reveal co-occurrence among bacterial communities (Supplementary Figure S4). Among the top 30 genera enriched in GC and GS, there was a strong positive correlation between genera and genera. Specifically in GS, it showed a closer correlation (GS: 332 edges, GC: 229 edges). *Paraburkholderia* and *Bacillus* in GS were positively correlated with 30 other genera. *Acidovorax, Citrobacter*, and *Bosea* in GC were positively correlated with more than 20 other genera. In GP, *Lactococcus* was negatively correlated with the other eight genera. This indicated that *Lactococcus* would probably impede the reproduction of these bacteria in GP processing. An *Enterobacter* genus, which was negatively related to it, was one of the most abundant genera in GP and had a positive correlation with the other 11 genera.

### Correlation of Bacterial Community and ARGs

**Figure 6:**
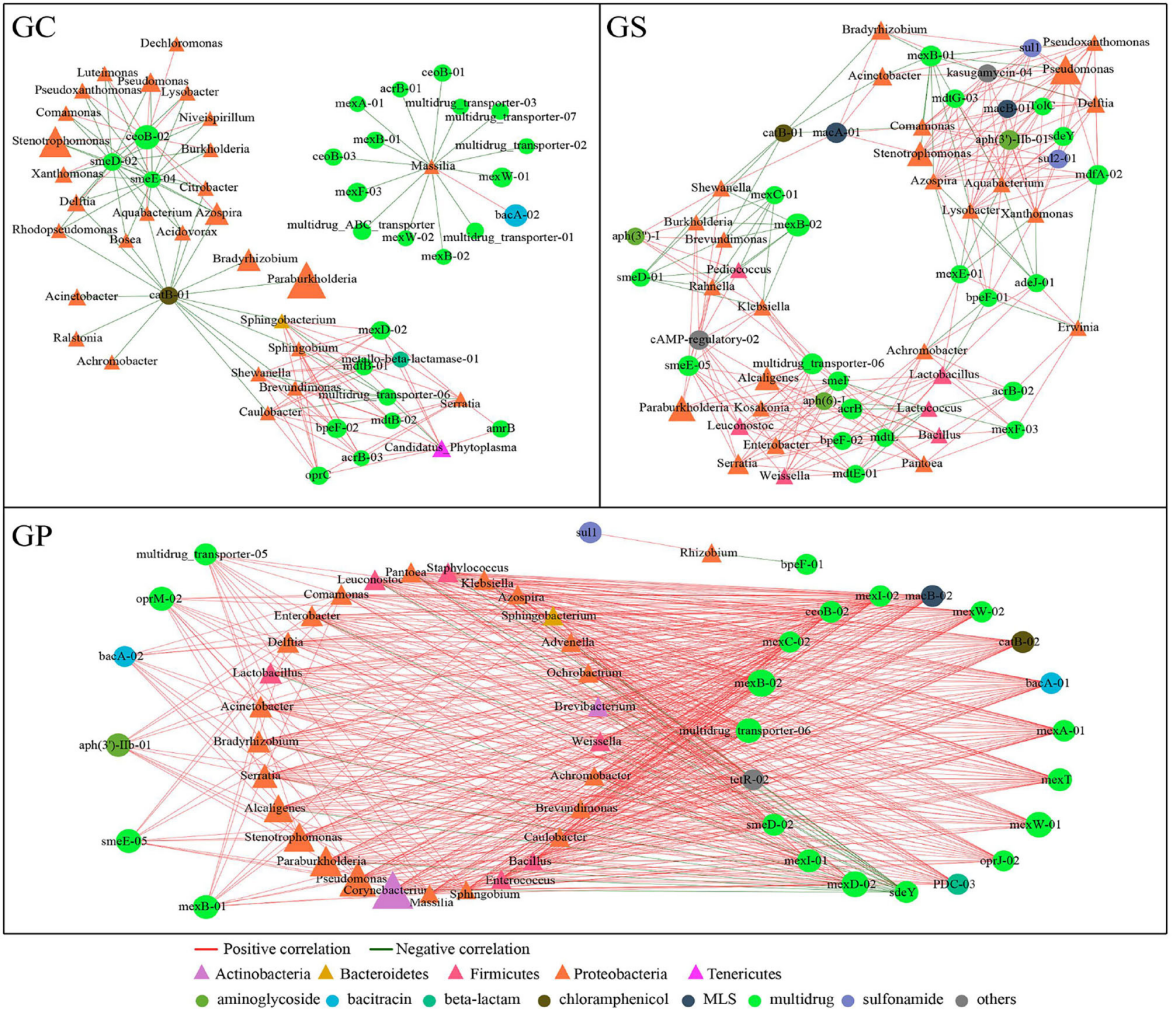
The network analysis revealing the co-occurrence patterns among ARGs and microbial taxa. The nodes were colored according to bacterial phyla and ARG types. Edges and node size weighted were based on the correlation coefficient and the relative abundance of ARGs and bacterial communities, respectively. The edge color represents a positive correlation (red) and a negative correlation (green).

Characteristics of ARG were significantly correlated with microbial composition and structure. Co-occurrence patterns among bacterial taxa and ARGs were therefore investigated by network analysis (Figure 6). A total of 58 nodes (30 microbial genera and 28 ARGs) and 136 edges, 57 nodes (30 microbial genera and 27 ARGs) and 541 edges, and 60 nodes (30 microbial genera and 30 ARGs) and 265 edges existed in GC, GS, and GP networks, respectively. If there was a strong (*r* > 0.90) and significant (*p* < 0.001) positive correlation among microbial groups and ARGs, the co-occurrence patterns might reveal a potential host of ARGs. In GC, in the view of *Candidatus*-*Phytoplasma* and *Serratia* were positively correlated with one beta-lactam and seven multidrug genes, which might be served as the possible hosts of these ARGs in GC. Besides, *Massilia* may be the host of ARGs and develop resistance to bacitracin (*bacA*). Relatively, the correlation profile among ARGs and bacterial taxa in GS was more complex. A single bacterial taxon correlated with diverse ARGs, and a single ARG correlated with different bacterial taxa. *Brevundimonas* might be the host of 22 kinds of ARGs. *Pseudomonas, Stenotrophomonas, Acinetobacter, Delftia*, etc. had a positive correlation with more than 20 kinds of ARGs and were resistant to multidrug, aminoglycoside, chloramphenicol, MLS, bacitracin, tetracycline, etc., indicating that these genera might be the important ARG hosts in GS. Although the bacterial communities between GS and GP were very similar, their association with ARGs was significantly different. In GP, *Brevundimonas* had a positive correlation with smeE-05 (multidrug) and aph (3”)-I (aminoglycoside). *Stenotrophomonas, Azospira, Aquabacterium*, and *Lysobacter* were positively correlated with 10 ARGs. *Lactobacillus* might be the host of acrB, mexE, mexF, mdtE, mdtL, etc. To sum up, in the process of GP processing, the correlation among bacterial communities and ARGs was significantly different under different processing processes.

## Discussion

Through high-throughput sequencing, metagenomics breaks the traditional microbial research method based on the culture. It can directly extract the DNA of the samples for operation and cover as much microbial genetic information contained in the sample with a big data sequencing library. This can not only accurately obtain information on the species composition and abundance of microorganisms in the sample, but also detect a large number of ARGs at the same time. This can also provide convenience for finding new genes, predicting the function of microorganisms, and studying the diversity of microorganisms (39). A large number of studies have confirmed that the extensive use of antibiotics can not only bring serious antibiotic residue problems but also make many bacteria obtain drug resistance. Other bacteria and even humans can become receptors for drug resistance gene migration (40), bringing the potential risk of drug resistance transmission. Based on metagenomics, this study investigated the microorganisms and drug resistance during GP processing and found that there were 43 ARGs, 123 ARGs, and 85 ARGs in GC, GS, and GP, respectively. Multidrug was the most abundant ARG in the three periods, bacitracin and chloramphenicol were the main ARGs in GC, while beta-lactam and aminoglycoside were the main ARGs in GS; sulfonamide, MLS, and aminoglycoside were the main ARG genes in GP. The average abundance of ARG in GC, GS, and GP was 21.0, 88.7, and 39.7, respectively. With the processing of GP, the performance of ARGs (especially multidrug) increased first and then decreased, which indicated that ARGs were diverse in food. D'costa et al. (12) have proved that antibiotic resistance can occur naturally. In addition, studies have shown that fertilization with manure or municipal sewage sludge will cause ARGs to spread to farmland and even harvested vegetables (17, 41). It is not strange to detect resistance genes in garlic and its products.

Most of the ARGs were recalcitrant during GP processing. During the processing of GP, the processing from GC to GS would increase the abundance of ARGs, indicating that deep processing might lead to the occurrence and spread of ARGs. With the further progress of processing, the abundance of ARGs showed different degrees of reduction during the process from GS to GP. Although the processing of GS has undergone heat treatment, the processing temperature and time at this time are not enough to eliminate ARGs (42, 43). Although the bacterial communities between GS and GP were very similar, it was not completely consistent, and their association with ARGs was significantly different, which may lead to the different abundance of Arg in GS and GP (44). In addition, the variations of nutrient resources, oxygen contact area, and sulfur-containing compounds lead to differences in bacterial communities.

Some studies pointed that the microbial community is an important factor in the construction of the target ARG or local resistance body (45). *Proteobacteria* was the dominant phylum in GC, GS, and GP, which is consistent with previous studies Previous studies have shown that *Proteobacteria* was the most abundant group in apple branches and tomatoes (46, 47), and *Proteobacteria* was also the dominant group in leeks, garlic, and onions (25). Consistent with the higher ARG abundance in GS, the relative abundances of *Alcaligenes, Serratia, Pseudomonas*, and *Corynebacterium* belonging to the *Proteobacteria* phylum were also significantly higher than those of GC and GP. This indicated that these genera might be the reason for the higher ARG enrichment. The diversity of ARGs in GS and GP was higher than that in GC, which might be because the common genera of GS and GP (*e.g., Pantoea, Leucanostoc, and Alcaligenes*) do not exist in GC, indicating that the prevalence of these genera might lead to the diversification of ARGs.

As a possible host of ARGs, microorganisms will multiply or decay under different conditions, thereby affecting the persistence and reproduction of ARGs (48). Network analysis can provide new ideas for ARGs and their potential hosts, enabling us to observe the complex relationship of microbial taxa and ARGs, which has proven to be a reliable method for exploring possible ARG hosts in complex environments (49). Since certain microbial groups contain specific ARGs, correspondingly similar abundance trends can be obtained. BacA-01, macB-02, and mexD-01 were the most closely connected nodes in GP, multidrug ABC transporter was the most closely connected node in GC, and mexD-01, mexW-02, mexB-02, multidrug transporter-06, mexI-02, and mexE-02 were tightly connected nodes in GS, indicating that these genes can be used as indicator genes of ARGs, and the co-occurrence of ARGs during GP processing can be analyzed qualitatively and quantitatively based on these genes (41). *Candidatus-Phytoplasma, Serratia*, and *Massilia* were possible hosts of ARGs in GC; the relationship of ARG and bacterial groups in GS was more complicated. *Brevundimonas, Pseudomonas, Stenotrophomonas, Acinetobacter*, and *Delftia* were possible hosts of ARGs in GS. The GS and GP groups share more genera and have very similar bacterial communities, but their associations with ARGs were significantly different. *Brevundimonas, Stenotrophomonas, Azospira, Aquabacterium, Lysobacter*, and *Lactobacillus* may be the hosts of ARGs in GP. These results showed that microorganisms were an important source of ARGs, which contribute to the accumulation of ARGs during GP processing. Under different treatment processes, the correlation of bacterial communities and ARGs was also different. It is necessary to make further efforts to probe into the specific relationships of microbial communities and ARGs and offer valuable facts for ARG control in GP processing.

## Conclusions

This study was based on metagenomic sequencing technology to investigate the microbial communities and antibiotic resistance of GC, GS, and GP. The study documented the diversity and abundance of ARGs, and also the high correlation of bacterial community compositions and ARGs during the GP processing. As the GP processing progresses, the performance of ARGs increased first and then decreased, and there was a higher similarity among ARGs in the middle and late stages of GP processing. The characteristics of ARGs were significantly related to the composition and structure of microorganisms. The GS and GP groups shared more genera (385), *Paraburkholderia, Stenotrophomonas, Bradyrhizobium*, and *Azospira* were the dominant genera in GC, *Paraburkholderia, Stenotrophomonas, Corynebacterium*, and *Pseudomonas* were the dominant genera in GS, and *Paraburkholderia, Stenotrophomonas, Pseudomonas*, and *Enterobacter* were the dominant genera in GP. *Proteobacteria, Actinobacteria*, and *Firmicutes* were the dominant gates. The potential indicators and hosts of ARGs were evaluated on the basis of the co-occurrence pattern of ARG subtypes and microbial taxa. In general, during the GP processing, the correlation of bacterial communities and ARGs were significantly different under various treatments. This study will provide insights into the use of microbial changes to manage and control the spread of ARGs during the GP processing and help to supervise and manage the production of garlic products.

## Data Availability Statement

The datasets presented in this study can be found in online repositories. The names of the repository/repositories and accession number(s) can be found in the article/Supplementary Material.

## Author Contributions

YL, CL, PG, YW, XW, and XL contributed to the study design, data collection, data analysis, wrote the first draft, and revised the manuscript. JS, JX, JS-G, and NL were the supervisor of the project and contributed to the study design, data analysis, and manuscript revision. All the authors reviewed and accepted the content of the final manuscript.

## Funding

This study was supported by the National Natural Science Foundation of China (31972000), the Taishan Scholars's Program of Shandong for JS and TaiShan Industrial Experts Programme (No. tscy20200121).

## Conflict of Interest

The authors declare that the research was conducted in the absence of any commercial or financial relationships that could be construed as a potential conflict of interest.

## Publisher's Note

All claims expressed in this article are solely those of the authors and do not necessarily represent those of their affiliated organizations, or those of the publisher, the editors and the reviewers. Any product that may be evaluated in this article, or claim that may be made by its manufacturer, is not guaranteed or endorsed by the publisher.

## Supplementary Material

Supplementary Figure S1 Number and distribution of shared resistance genes detected during GP processing.

Supplementary Figure S2 Heatmap of the relative abundance of ARGs during GP processing. The color transition from dark blue to dark red represents the relative abundance from low to high.

Supplementary Figure S3 (Left) Venn diagram for each out to compare the richness shared among three groups. (Right) PCA based on the Bray–Curtis distance showing the overall distribution pattern of bacterial communities during GP processing.

Supplementary Figure S4 Network analysis revealing the co-occurrence pattern among bacterial communities in GC, GS, and GP samples. The nodes were colored according to the bacterial phyla. A connection represents a strong (Spearman's correlation coefficient *r* > 0.90) and significant (*p* < 0.01) correlation. Edges and node size weighted were based on the correlation coefficient and the relative abundance of bacteria, respectively. The edge colors represent positive correlation (red) and negative correlation (green).
