# Supplementary figures and images for: The Formation of Antibiotic Resistance Genes in Bacterial Communities During Garlic Powder Processing
Source: Front Nutr. 2021 Dec 16;8:800932. doi: 10.3389/fnut.2021.800932 (PMC8717741; doi:10.3389/fnut.2021.800932)

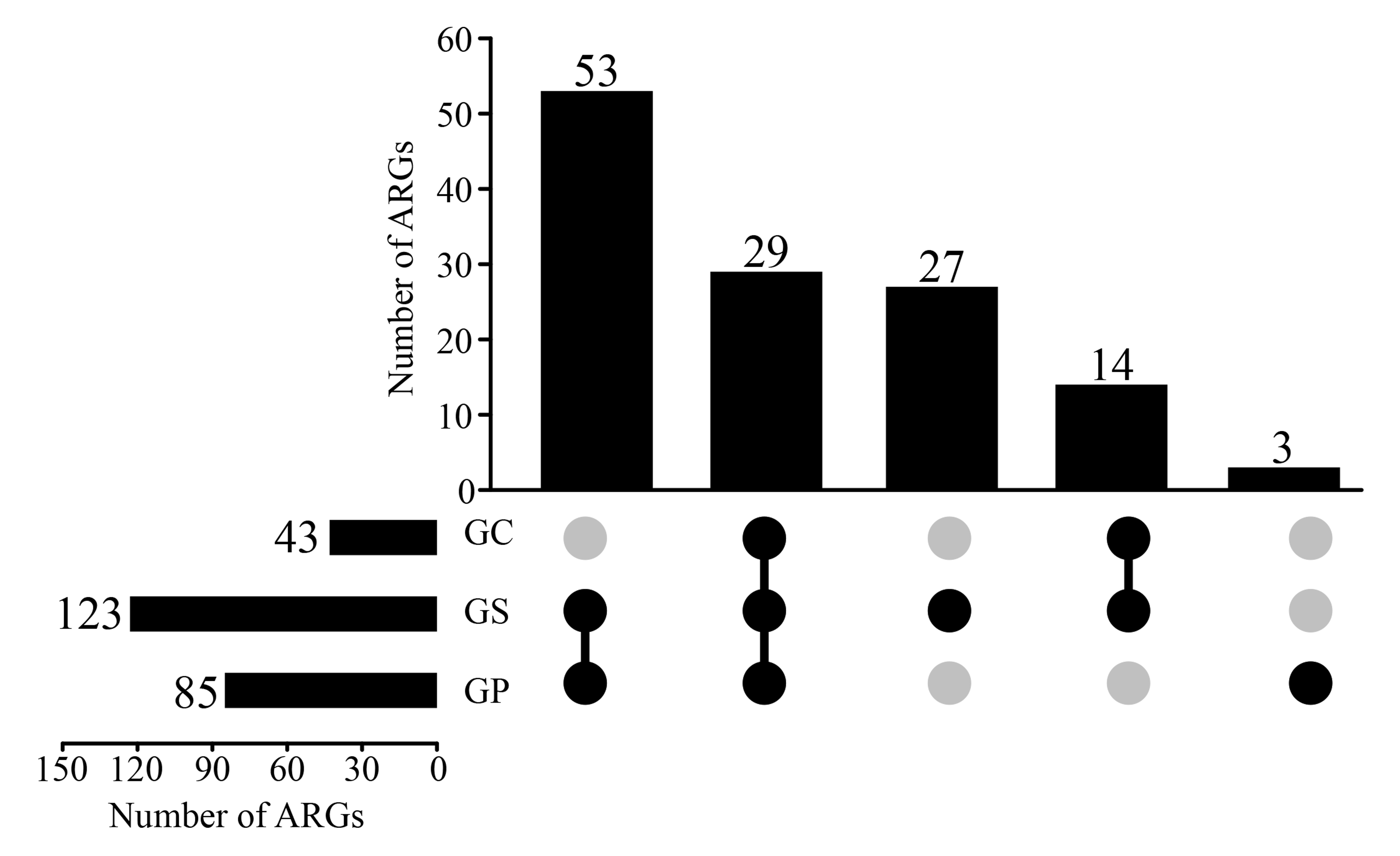

Supplement: Supplementary Figure S1 — Number and distribution of shared resistance genes detected during GP processing. [file Image_1.tif]

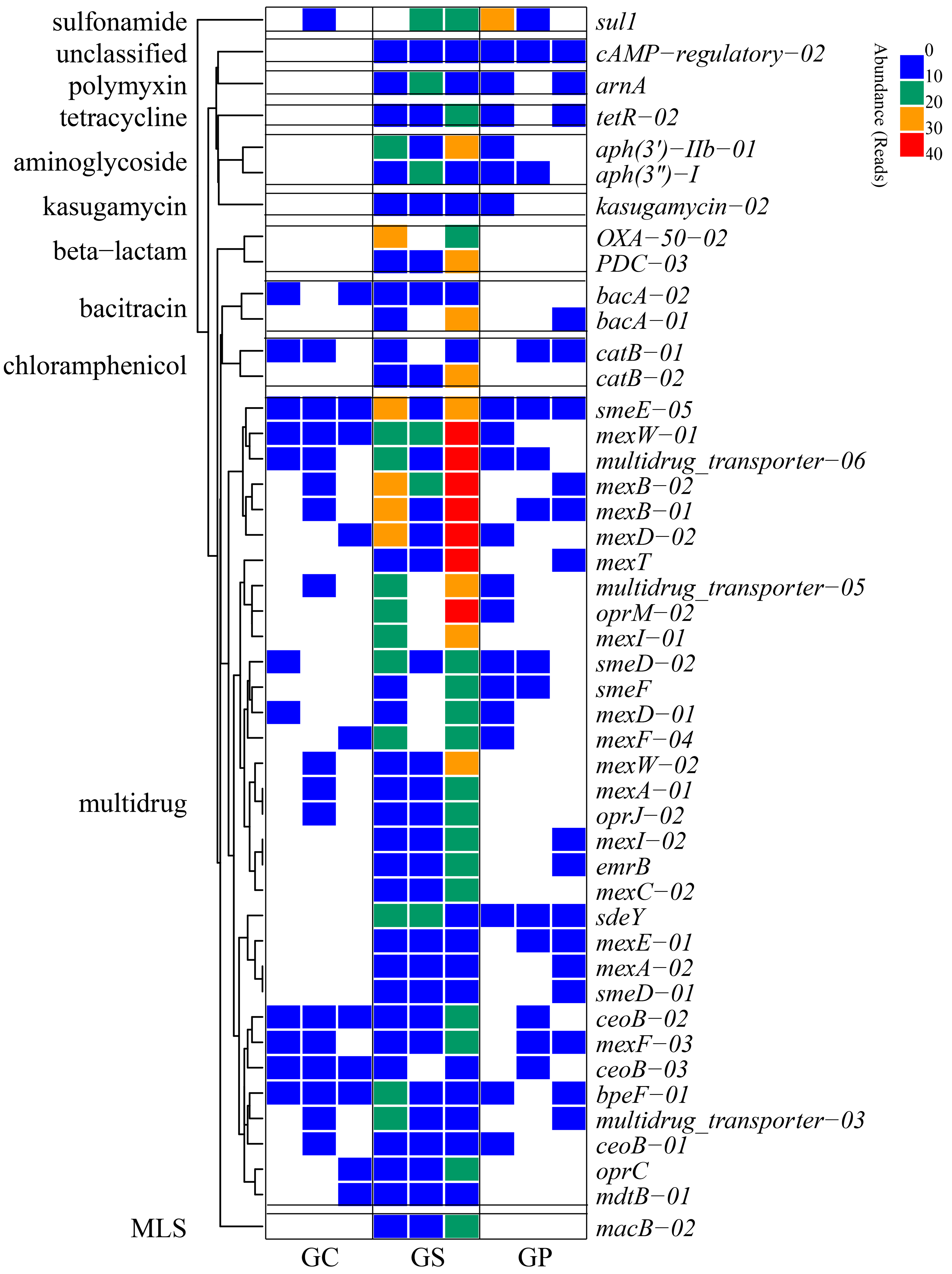

Supplement: Supplementary Figure S2 — Heatmap of the relative abundance of ARGs during GP processing. The color transition from dark blue to dark red represents the relative abundance from low to high. [file Image_2.tif]

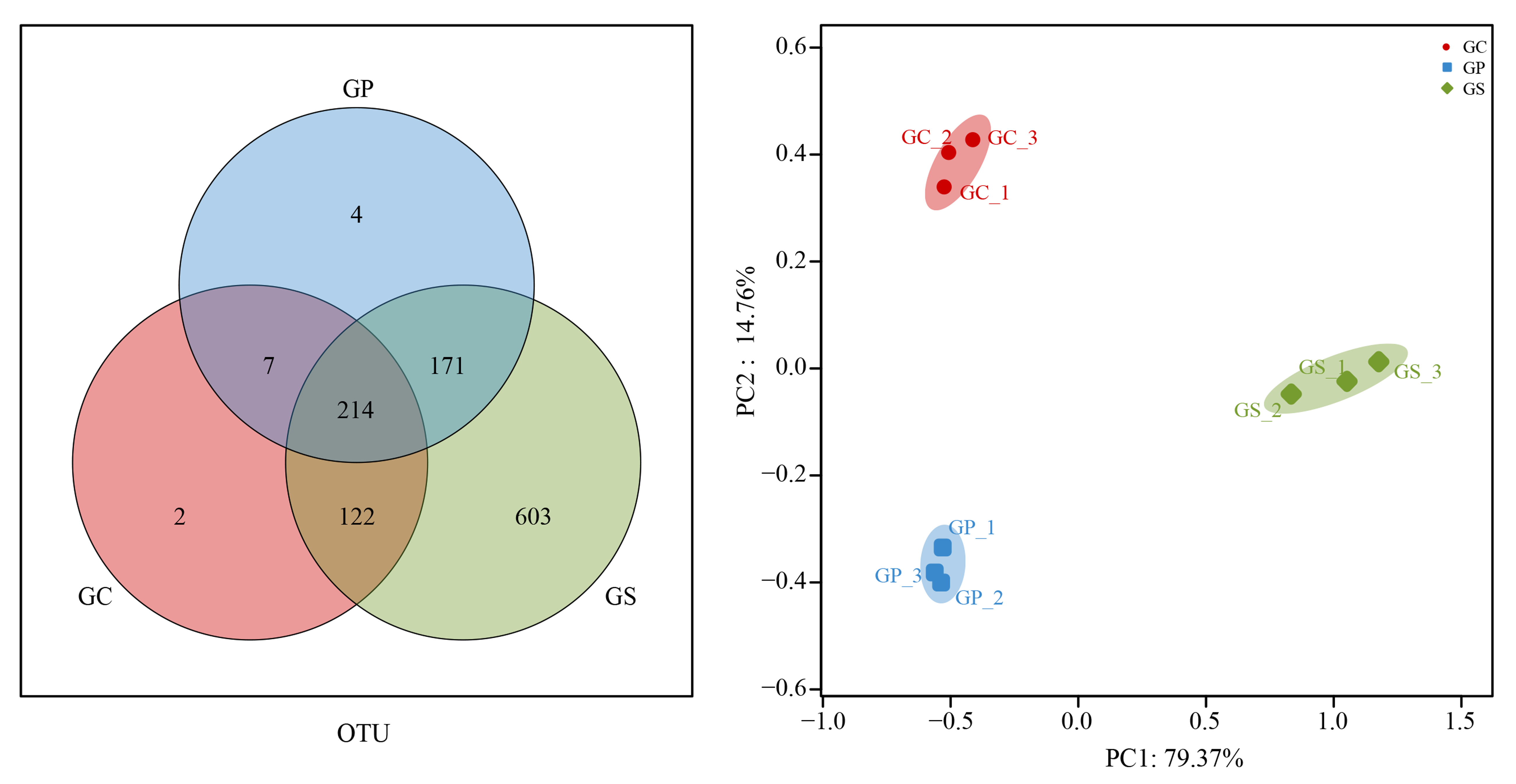

Supplement: Supplementary Figure S3 — (Left) Venn diagram for each out to compare the richness shared among three groups. (Right) PCA based on the Bray–Curtis distance showing the overall distribution pattern of bacterial communities during GP processing. [file Image_3.tif]

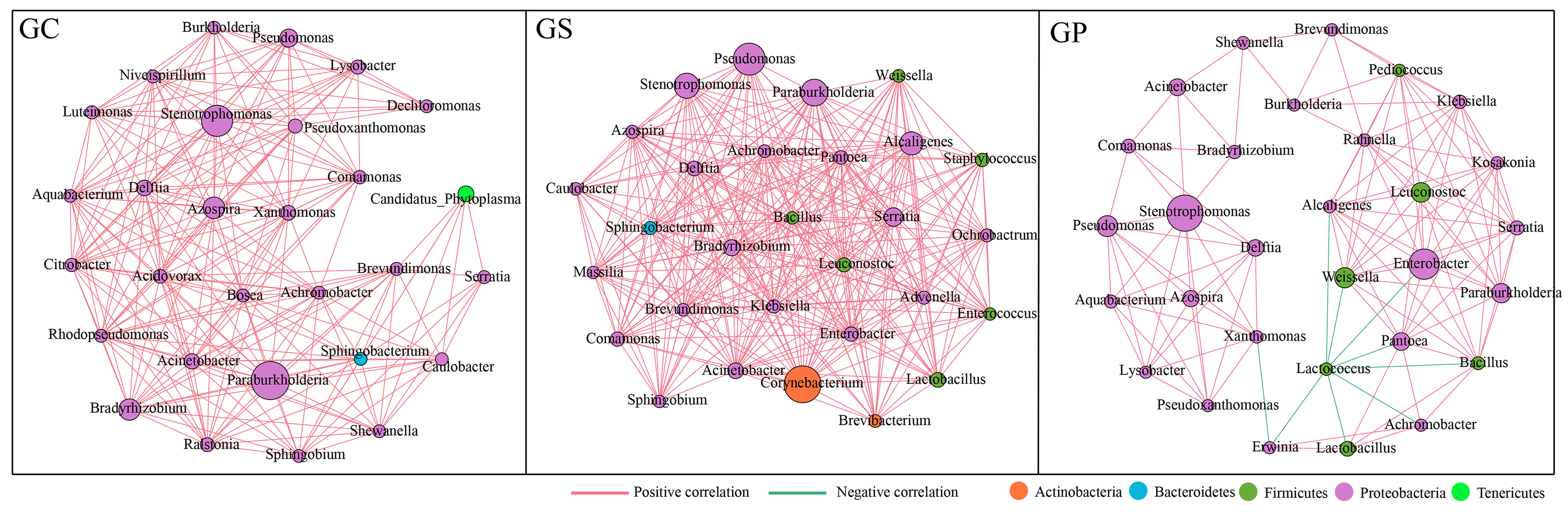

Supplement: Supplementary Figure S4 — Network analysis revealing the co-occurrence pattern among bacterial communities in GC, GS, and GP samples. The nodes were colored according to the bacterial phyla. A connection represents a strong (Spearman's correlation coefficient r > 0.90) and significant (p < 0.01) correlation. Edges and node size weighted were based on the correlation coefficient and the relative abundance of bacteria, respectively. The edge colors represent positive correlation (red) and negative correlation (green). [file Image_4.tif]
